# Primary leiomyosarcoma with osteosarcomatous differentiation of the breast

**DOI:** 10.4322/acr.2024.476

**Published:** 2024-02-26

**Authors:** Ekta Sethi, Sunayana Misra, Arvind Ahuja

**Affiliations:** 1 Atal Bihari Vajpayee Institute of Medical Sciences and Dr. Ram Manohar Lohia Hospital, Department of Pathology, New Delhi, India; 2 Sir Ganga Ram Hospital, Histopathology Department, New Delhi, India

**Keywords:** Breast, Leiomyosarcoma, Mastectomy, Sarcoma

## Abstract

Primary leiomyosarcoma with osteosarcomatous differentiation of the breast is an uncommon entity. We present the case of a 37-year-old female who presented with a lump in the breast and pulmonary lesions on PET-CT, for which she underwent a toilet mastectomy. Histopathological examination revealed a tumor with cells arranged in sheets. These tumor cells had moderate eosinophilic cytoplasm, highly pleomorphic, irregular hyperchromatic nuclei, coarse chromatin, and prominent nucleoli. Areas with spindle-cell morphology were noted. Osteoid was seen intermingling with the tumor along with numerous osteoclast-like multinucleate giant cells. A wide panel of Immunohistochemistry was applied, and Desmin, h-Caldesmon, SMA, and Vimentin were positive. The patient died 3 months post-surgery and had a recurrence at the surgical site.

## INTRODUCTION

Primary sarcomas of the breast are rare lesions accounting for 1% of all malignant breast tumors and less than 5% of all soft tissue sarcomas.^1,2^ Angiosarcoma is the most common mammary sarcoma. Other relatively common sarcomas include liposarcomas, malignant fibrous histiocytomas, fibrosarcoma, and leiomyosarcomas.^3^ Here, we present a case of a 37-year-old female who underwent mastectomy and, on histopathological examination, turned out to be a rare case of leiomyosarcoma (LMS) with osteosarcomatous differentiation along with pulmonary metastasis.

## CASE REPORT

**Figure 1:**
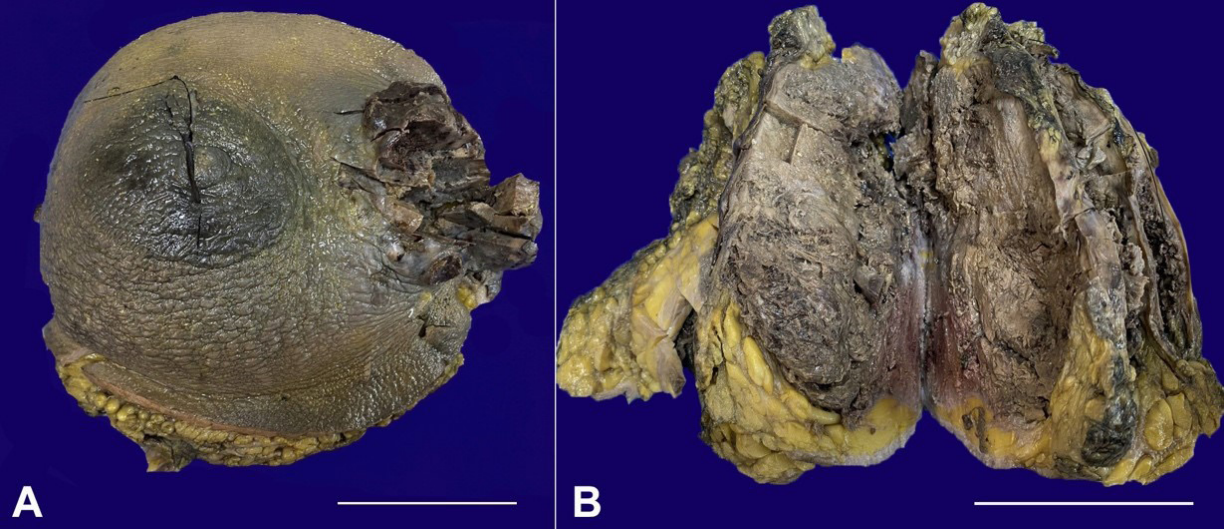
Gross view of the surgical specimen. **A -** Left mastectomy specimen showing a fungating ulcer on the lateral aspect (scale bar= 10 cm); **B -** Cut section shows a large fleshy dark brown to tan tumor occupying almost the entire breast (scale bar = 10 cm).

A 37-year-old female presented to the Surgical outpatient department complaining of an increasing left breast lump for one year. The lesion presented with overlying skin ulceration and bleeding over the last month, accompanied by dull, aching pain with gradual worsening. There was no history of nipple retraction, discharge, or similar swelling in the opposite breast. On examination, the left breast was diffusely enlarged, and a lump was present in the upper and lower outer quadrants with ulceration and bleeding. The mass was hard and fixed over the chest wall. Multiple lymph nodes were palpated in the left axilla. PET CT revealed a solitary pulmonary nodule in the apical segment. The patient was subjected to a lumpectomy over the left breast, in an outside hospital following which she received 5 cycles of chemotherapy (anthracyclin and taxane with cyclophosphamide), to which she did not respond. She then underwent a left toilet mastectomy with split-thickness skin grafting from the right thigh in our hospital. The left mastectomy specimen showed a polypoidal fungating ulcerated growth on gross examination. On serial slicing of the breast, a large variegated tumor with solid cystic and hemorrhagic appearance measuring 17×17×8 cm was noted, involving the skin and almost the whole breast (Figure 1).

**Figure 2:**
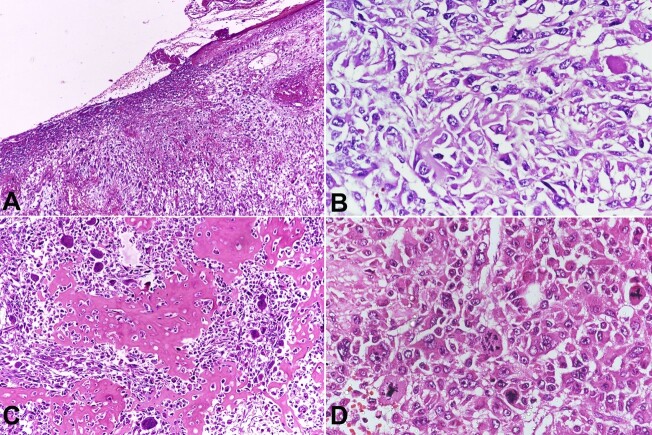
Photomicrographs of the tumor. **A -** Tumor arranged in diffuse sheets with overlying ulcerated skin (H&E,10x); **B -** Tumor cells with moderate eosinophilic cytoplasm, pleomorphic nuclei, coarse chromatin and prominent nucleoli (H&E,40x); **C -** Numerous osteoclast type giant cells intermingling with areas of osteoid (H&E,20x); **D -** Atypical mitotic figures (H&E, 20x).

Histologically, the tumor was highly cellular. The tumor cells were arranged in diffuse sheets (patternless pattern). These cells were highly pleomorphic and had irregular hyperchromatic nuclei, coarse chromatin, prominent nucleoli, and a moderate eosinophilic cytoplasm. Areas with spindle-cell morphology were seen. Osteoid formation was seen intermingling with the tumor and numerous osteoclast-like multinucleate giant cells (OGC). Many atypical mitotic figures and large areas of necrosis and hemorrhage were seen. No glandular component was present in any of the sections after extensive sampling. The tumor had directly invaded the skeletal muscle, dermis, and epidermis, which was ulcerated. Lymphovascular emboli were also present (Figure 2).

Given the morphological diagnosis of sarcoma, a large immunohistochemistry (IHC) panel was applied to reach a definitive diagnosis (Table 1).

**Table 1:** Immunohistochemistry panel

Marker	Clone	Interpretation	Marker	Clone	Interpretation
Desmin	D33	Positive	Myogenin	MyG007	Negative
SMA	1A4	Positive	MyoD1	EP212	Negative
Vimentin	V9	Positive	S100	15E2E2	Negative
H-Caldesmon	h-CALD	Positive	CD10	56C6	Negative
PanCK	LU-5	Negative	CD117	EP10	Negative
EMA	E29	Negative	β-HCG	M94138	Negative
P63	4A4	Negative	ER	EP1	Negative
34βE12	34βE12	Negative	PR	EP2	Negative
CD34	QBEnd-10	Negative	Her2neu	SP101	Negative
CD31	BC2	Negative	Ki67	MIB7	15%

**Figure 3:**
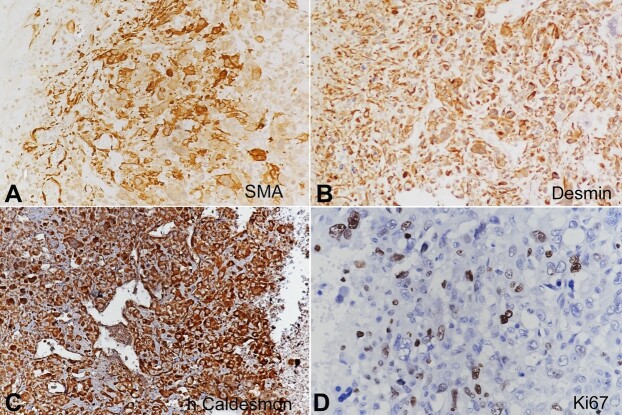
Photomicrographs of the tumor. Immunohistochemical stains. **A -** positive Smooth Muscle Actin (20x); **B -** positive Desmin (20x); **C -** positive h-Caldesmon (20x); **D -** Ki67 -15% (20x).

Desmin, Vimentin, and h-Caldesmon were strongly positive in tumor cells, and SMA showed patchy strong positivity. The Ki67 proliferation index was 15% (Figure 3) PanCK, EMA, p63, 34βE12, CD34, CD31, Myogenin, CD10, CD117, S100, β-HCG, ER, PR and HER2neu were negative.

The histological and immunohistochemical findings favored the diagnosis of LMS with osteosarcomatous differentiation. Three months post-surgery, the patient expired with a recurrence of the tumor at the surgical site.

## DISCUSSION

Breast LMS is occasional, and primary LMS with osteosarcomatous differentiation is an even rarer malignant lesion.^2,3^ LMS with osteosarcomatous differentiation was first reported in the leg by Mentzel and Fletcher.^4^ Later, the lesion was reported in various other sites like the retroperitoneum, thigh, leg, forearm, mesentery, mediastinum, and back.^4-10^ After a thorough literature search through PubMed databases, only one case was diagnosed previously. Details of previously diagnosed primary LMS with osteosarcomatous differentiation of the breast have been described in Table 2.

**Table 2:** Leiomyosarcoma with osteosarcomatous differentiation in the literature

**Ref**	**Age/sex**	**Primary site**	**Local recurrence**	**Metastasis**	**Status**
Mentzel and Fletcher^4^	71/ M	Leg	Yes	Lung	Death after 22 months
Grabellus et al.^5^	35/ F	Groin	Yes	Lung	Unknown
Chen et al.^6^	66/ M	Retroperitoneum	Yes	Liver	Death after 8 months
Feeley et al.^7^	60/ M	Thigh	Yes	Occipital and lung	Unknown
Gaeta et al.^8^	65/ M	Forearm	Yes	Lung	Death after 23 months
Yu and Hornick^9^	32/ F	Retroperitoneum	No	Kidney, adrenal, lung and colon	Alive with no disease (15 years follow up)
Yu and Hornick^9^	65/ F	Retroperitoneum	No	Lung, colon, jejunum, ileum	Death after 5 months
Yu and Hornick^9^	66/ M	Mesentery	No	Liver, colon	Death after 2 months
Yu and Hornick^9^	44/ F	Rectum	n/a	n/a	n/a
Yu and Hornick^9^	59/ M	Mediastinum	n/a	n/a	n/a
Galama et al.^10^	87/F	Breast	No	No	Alive with no disease (2 years follow up)
CC	37/ F	Breast	Yes	Lung	Death after 3 months

CC = current case; F = female; M = male; Ref = reference; n/a = not applicable.

LMS of the breast most probably originates from the smooth muscle of the lactiferous ducts or blood vessels. It can also arise from the erector pili muscle seen in the areola.^3^ They usually present as well-circumscribed lesions with a mean size of 5.6 cm and are usually found in post-menopausal women.^1,3^ Clinically, they resemble malignant phyllodes tumor. It is difficult to diagnose this tumor by fine needle aspiration cytology, and it is usually diagnosed in resected specimens confirmed by IHC (SMA and Desmin positivity and negativity for epithelial markers).^2^

Dedifferentation is a well-known occurrence in certain types of cancer, such as liposarcoma and chondrosarcoma; however, in LMS, this phenomenon is rarely reported. In cases where dedifferentiation does occur in LMS, it typically manifests as a distinct transition from well-differentiated smooth muscle cells to a high-grade undifferentiated pleomorphic morphology resulting in loss of smooth muscle characteristics.^5,8^

Distinguishing between pleomorphic LMS and dedifferentiated LMS can be challenging, especially when there is an abrupt change in morphology but no corresponding shift in immunophenotype. It is believed that pleomorphic LMS and dedifferentiated LMS may represent different points on a histologic spectrum, with LMS transforming into a high-grade undifferentiated pleomorphic sarcoma (UPS).^6^

Leiomyosarcoma can also exhibit heterologous elements, such as metaplastic bone and cartilage. In some cases, there may be heterologous osteosarcomatous differentiation, characterized by malignant osteoid (bone-like) tissue and prominent osteoclastic giant cells. Dedifferentiated LMS of soft tissues is known to be an extremely aggressive tumor. According to the literature, these tumors have a mortality rate ranging from 50-65.2% and an incidence of metastasis as high as 89%. The loss of myogenic markers (markers associated with muscle differentiation) in LMS has been identified as a significant prognostic factor, contributing to its higher level of aggressiveness.^6^ Therefore, extensive sampling is recommended in LMS to capture the various components of the tumor.^9^

Osteoclastic giant cells, which are found in various soft tissue tumors, are characteristic features of certain fibrohistiocytic tumors, either benign or malignant. These include giant cell tumors of soft tissue (low malignant potential) and UPS with giant cells (high malignant potential). These tumors occasionally demonstrate metaplastic bone, which is not commonly observed in LMS. Heterotopic bone formation is also frequently seen in other soft tissue tumors like synovial sarcoma and malignant peripheral nerve sheath tumors (MPNST). However, the presence of malignant neoplastic osteoid or bone formation within a soft tissue tumor would typically lead to a diagnosis of extraskeletal osteosarcoma.^5^

After reviewing the literature, we found 11 previously reported extrauterine cases of LMS with osteosarcomatous differentiation (Table 2).

Considering all the reported cases, the average age of the patients was 57 years, with an equal gender ratio (1:1). Six patients had local recurrences and developed distant metastases despite aggressive treatment; in two cases, recurrence was unknown. In six patients, the outcome was fatal (death at two, three, five, eight, 22, and 23 months after surgical excision) due to the progression of the disease, while no clinical follow-up was detailed in four patients.

Microscopically, the differential diagnosis of LMS with osteosarcomatous differentiation includes osteosarcoma, UPS, pleomorphic LMS, pleomorphic rhabdomyosarcoma, metaplastic carcinoma, myoepithelial carcinoma, malignant phyllodes tumor with heterologous elements, MPNST, angiosarcoma, choriocarcinoma and metastasis.^11,12^ Therefore, a large panel of IHC is crucial to reach a correct diagnosis, prognostication, and management of the patients.

LMS are treated primarily with surgery. Chemotherapy and radiotherapy are not said to improve outcomes in these patients. Axillary dissection is not required as lymph node metastasis is rare. LMS with osteosarcomatous differentiation are aggressive tumors with poor prognosis, as evidenced by the recurrence and metastasis within a short span of post-surgery follow-up in most of the patients in the literature review.

## CONCLUSION

To conclude, we report the second case of LMS with osteosarcomatous differentiation of the breast. It is a sporadic, highly aggressive tumor that can be diagnosed by histopathology examination with a large panel of IHC to differentiate from the other malignant tumors that come in the list of differential diagnoses.
